# Bioprinted research models of urological malignancy

**DOI:** 10.1002/EXP.20230126

**Published:** 2024-02-20

**Authors:** Guanyi Wang, Xiongmin Mao, Wang Wang, Xiaolong Wang, Sheng Li, Zijian Wang

**Affiliations:** ^1^ Department of Urology Cancer Precision Diagnosis and Treatment and Translational Medicine Hubei Engineering Research Center Zhongnan Hospital of Wuhan University Wuhan China; ^2^ Department of Biomedical Engineering and Hubei Province Key Laboratory of Allergy and Immune Related Disease TaiKang Medical School (School of Basic Medical Sciences) Wuhan University Wuhan China; ^3^ Lewis Katz School of Medicine Temple University Philadelphia Pennsylvania USA

**Keywords:** bioprinting, interdisciplinary science, research model, tumor, urology

## Abstract

Urological malignancy (UM) is among the leading threats to health care worldwide. Recent years have seen much investment in fundamental UM research, including mechanistic investigation, early diagnosis, immunotherapy, and nanomedicine. However, the results are not fully satisfactory. Bioprinted research models (BRMs) with programmed spatial structures and functions can serve as powerful research tools and are likely to disrupt traditional UM research paradigms. Herein, a comprehensive review of BRMs of UM is presented. It begins with a brief introduction and comparison of existing UM research models, emphasizing the advantages of BRMs, such as modeling real tissues and organs. Six kinds of mainstream bioprinting techniques used to fabricate such BRMs are summarized with examples. Thereafter, research advances in the applications of UM BRMs, such as culturing tumor spheroids and organoids, modeling cancer metastasis, mimicking the tumor microenvironment, constructing organ chips for drug screening, and isolating circulating tumor cells, are comprehensively discussed. At the end of this review, current challenges and future development directions of BRMs and UM are highlighted from the perspective of interdisciplinary science.

## Graphical abstract

In this review, a series of bioprinted research models are presented for the fundamental research of urological malignancies. This review summarizes and discusses five aspects, including bioprinting technology, polymer‐based inks, collection of seeding cells, culturing of cancer models, and the applications for biomedical research. Moreover, several current challenges and future directions are prospected and highlighted.

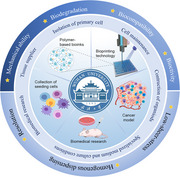

## 1 INTRODUCTION

Urological malignancies (UMs), including renal carcinoma, urothelial cancer, prostate cancer, testicular cancer and penile cancer, pose a worldwide threat to human health.^[^ ^1^ ^]^ More than 2 million cases of newly diagnosed UM and approximately 0.8 million deaths from UM were reported in 2020.^[^ ^2^ ^]^ In particular, prostate cancer (PCa) is one of the most common cancers in men, accounting for 27% of all cancer cases in men.^[^ ^3^ ^]^ Researchers have given much attention to clinical research focusing on early diagnosis and precision treatment. For example, the detection of prostate‐specific antigen (PSA) provides valuable information for decision‐making in PCa and thus is recommended by many clinical guidelines.^[^ ^4^ ^]^ The application of laparoscopy and Da Vinci surgical robots has been extensively promoted, and a series of minimally invasive operations have been proposed. In the past few years, tumor‐targeted therapy (TTT) has been of increasing interest.^[^ ^5^ ^]^ Several drugs, such as erdafitinib and sorafenib, have been approved by the Food and Drug Administration (FDA), substantially extending the survival time of patients with locally advanced and metastatic UM.

Basic research on UM has recently achieved significant progress in understanding tumor biology and basic discoveries that could change clinical approaches.^[^ ^6^ ^]^ Moreover, several key questions and breakthroughs, including the identification of novel cancer vulnerabilities, the maturation of immunotherapy in the era of precision medicine, an improved understanding of the tumor microenvironment (TME), genomics‐guided cancer precision medicine, and the relationship of cancer and the microbiome, were highlighted as a roadmap for the next decade.^[^ ^7^ ^]^ The scientific hypotheses are being addressed one by one. Moreover, the scope of fundamental research is rapidly expanding, creating unprecedented demand for advanced research models. Classical 2D cell culture cannot imitate the main characteristics of primitive tumors in vivo; thus, the experimental results diverge from the real world to varying degrees.^[^ ^8^ ^]^ Basic research on UM has recently spurred innovations in research models. Researchers have established a series of alternative research models, including primary cell models, tumor‐bearing models, and drug‐induced tumor models.^[^ ^9^ ^]^ However, a 3D‐bioprinted research model of UM with good biomimetics and ease of use is still lacking.

Bioprinting is a viable method for establishing 3D research models. In bioprinting, a computer‐assisted platform deposits and assembles polymer‐based inks (PBIs) to fabricate constructs with or without seeding cells.^[^ ^10^ ^]^ A series of bioprinting techniques, including extrusion‐based bioprinting, drop‐based bioprinting, laser projection‐based 3D bioprinting, direct writing bioprinting, magnetic 3D bioprinting and acoustic droplet bioprinting, have been investigated. The cells can be seeded onto or inside the constructs to mimic heterogeneous 3D structures such as tumors in vivo, resulting in great advantages over 2D cell culture.^[^ ^11^ ^]^ The biocompatibility and bioactivities of PBIs are vital for determining the fate of the seeded cells.^[^ ^12^ ^]^ Due to their high customization, precision and repeatability, bioprinted research models (BRMs) may be a powerful tool to bridge the gap between fundamental research and real‐world applications.

BRMs have been extensively reported, especially in UM. For example, Kim et al. established a bioprinted bladder cancer chip to model immunotherapy response.^[^ ^13^ ^]^ Bioprinting can be combined with other frontier techniques; for example, bioprinting can be combined with induced pluripotent stem cells (iPSCs) to generate bone, neural, and vascular tissues with self‐renewal and differentiation potential; with patient‐derived xenografts (PDXs) to establish tumor organoids; and with microfluidics to screen new drugs. The properties of PBIs can be physically and chemically modified to prepare multifunctional constructs. For example, metal and metal oxide (MMO) nanoparticles such as Ag and ZnO can be incorporated into PBIs to confer antibacterial activities.^[^ ^14^ ^]^ Notably, bioprinting has achieved single‐cell resolution, significantly improving its application potential.^[^ ^15^ ^]^

**FIGURE 1:**
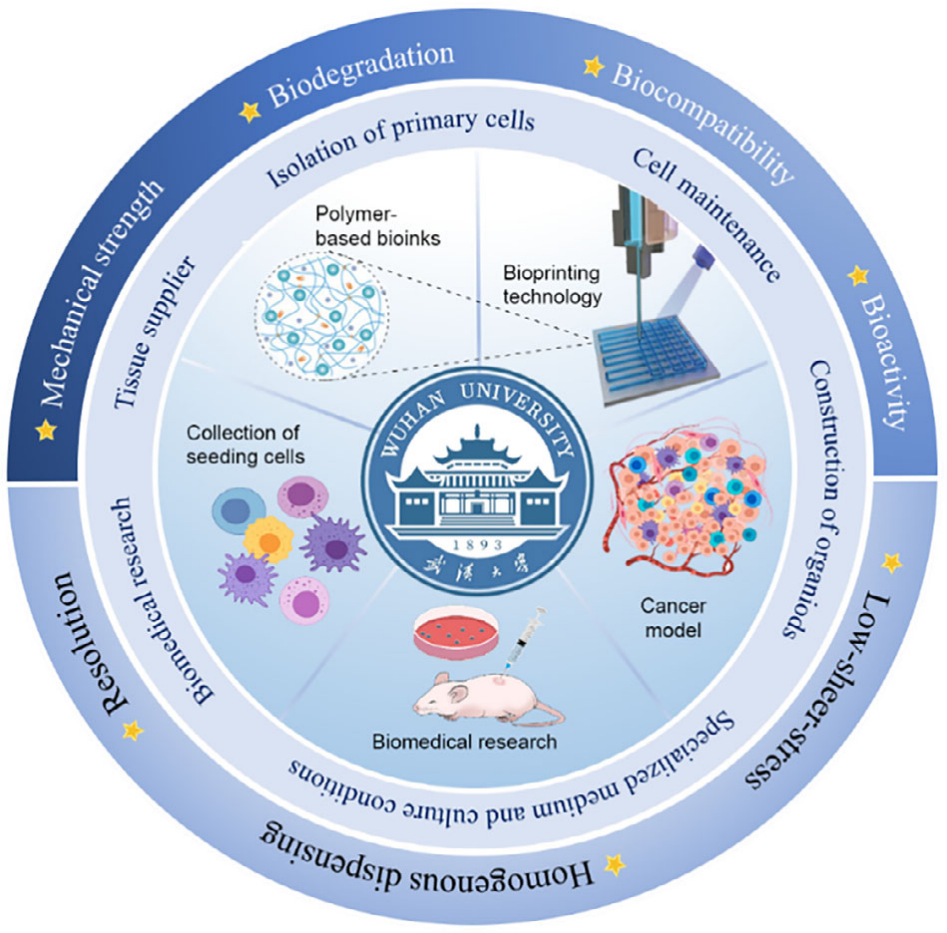
State‐of‐art diagram of bioprinted research models in urological malignancies. This review discusses five aspects, including bioprinting technology, polymer‐based inks, collection of seeding cells, culturing of cancer models and the applications for biomedical research. To establish ideal BRMs, a series of key parameters, such as mechanical strength, biocompatibility, bioactivity and biodegradation of polymer‐based inks are addressed and marked with an asterisk.

A scheme of this review is shown in Figure 1. We first compare various research models of UM and then summarize the bioprinting techniques that are capable of fabricating BRMs. After that, recent advances in these BRMs are presented. This review focuses on five aspects: bioprinting technology, polymer‐based inks, the collection of seeding cells, the culture of cancer models, and applications for biomedical research. Several key points are discussed and highlighted. Finally, current challenges and future directions are presented. This review aims to clarify the novelty and importance of BRMs in UM, thereby providing valuable insights for fundamental research, oncology and the pharmaceutical industry.

## 2 RESEARCH MODELS IN UROLOGICAL MALIGNANCY

### 2.1 Urological research models are abundant

In recent decades, researchers have developed various research models in vivo and in vitro for fundamental research on UM. Herein, existing research models are summarized and listed in Table 1. They are abundant in type and can be divided into eight categories, including cell lines, induced pluripotent stem cells (iPSCs), conditionally reprogrammed cells (CRCs), organoids, spontaneous tumors, induced primary tumors, genetically engineered models (GEMs), and xenograft models. These models have been extensively used for exploring tumorigenesis, tumor maintenance and therapeutic susceptibility. However, it is still challenging to model the genetic heterogeneity and lineage specificity of UM and to mimic the characteristics of tumor growth and the tumor‐specific microenvironment.^[^ ^16^ ^]^

**TABLE 1:** Research models in urological malignancies.

	Clinical characterization			Research models in vitro				Research models in vivo				
											Xenograft models	
	Pathological type	Incidence (%)	Mortality (%)	Cell lines	iPSCs	CRC	Organoids	Spontaneous tumor	Induced tumor	GEM	CDX	PDX
**Renal cancer**	ccRCC, chRCC, pRCC,	2.2	1.8	769‐P, 786‐O, A498, ACHN, CAKI‐1, CAKI‐2, OS‐RC‐2	Hereditary PRCC,^[^ ^17^ ^]^ VHL syndrome^[^ ^18^ ^]^	ccRCC, MCC/CDC,^[^ ^19^ ^]^	ccRCC, pRCC, chRCC	CD‐1 Mice (M/F: 0.13%), B6C3F1 Mice (F: 0.16%)^[^ ^20^ ^]^ CE Mice (M: 100%/F: 79%), NH Mice (M/F: 10%)	BBN (SD/cShi rats),^[^ ^21^ ^]^ TBA (Fischer 344 rats/B6C3F1 rats)^[^ ^22^ ^]^ nitrosamines; potassium bromate; Fe‐NTA; d‐limonene; tetrachloroethylene; hydroquinone (rats/mice)^[^ ^23^ ^]^	Vhl^−/−^Pbrm1^−/−^ (Ksp‐Cre),^[^ ^24^ ^]^ Vhl ^−/−^Pbrm1^−/−^ (Pax8‐Cre),^[^ ^25^ ^]^ Vhl^−/−^ Bap1^+/−^ (Pax8‐Cre),^[^ ^25^ ^]^ Vhl^−/−^ Cdkn2a^−/−^ (Ksp‐Cre+Myc),^[^ ^26^ ^]^ Vhl^−/−^Trp53^−/−^Rb1^−/−^ (Ksp‐Cre)^[^ ^27^ ^]^	CAKI‐1/A498/786‐O (Nu/Nu),^[^ ^28^ ^]^ CAKI‐1/A498 (BALB/c‐nu/nu),^[^ ^29^ ^]^	MDA‐RCC‐48 (BALB/c nude),^[^ ^30^ ^]^ 1∼13 (RAG2^−/−^ γC^−/−^),^[^ ^31^ ^]^ RP‐R‐02 (SCID),^[^ ^32^ ^]^ 1−94 (NOD‐SCID),^[^ ^33^ ^]^ ccRCC (NSG)^[^ ^34^ ^]^
**Bladder cancer**	UC, SCC, ADENO‐CA, SmCC	3.0	2.1	5637, BIU87, EJ, T24, RT112, RT4	T24 transduced with 4Fs,^[^ ^35^ ^]^	UDC,^[^ ^36^ ^]^ HG‐UC SmCC,^[^ ^37^ ^]^ CRC269 CRC269‐R CRC293 CRC382^[^ ^38^ ^]^	PDBTOL,^[^ ^39^ ^]^ MIBC,^[^ ^40^ ^]^ OBHUC^[^ ^41^ ^]^	Dogs invasive TCC,^[^ ^42^ ^]^ K9TCC (1 Lillie; 2 Dakota; 4 Molly; 5 Lilly) cell lines from dogs,^[^ ^43^ ^]^ p53 null mice^[^ ^44^ ^]^ BN rats (M: 35%)	BBN, FANFT, MNU (Rodents)^[^ ^45^ ^]^	Transgenic models (Uroplakin‐II Promoter^[^ ^46^ ^]^; Cytokeratin 19^[^ ^47^ ^]^; Uroplakin‐II^[^ ^48^ ^]^), Cre drivers,^[^ ^49^ ^]^ delivery of Adeno‐Cre^[^ ^50^ ^]^	MBT2 cells (C3H/He mice),^[^ ^51^ ^]^ MB49 cells (C57BL/6 mice),^[^ ^52^ ^]^ UMUC1/3/13 cells (Nude)^[^ ^53^ ^]^	CoCaB1 (CB‐17 SCID),^[^ ^40a^ ^]^ BL0269F (NSG),^[^ ^54^ ^]^ LTL392 (NOD‐ SCID)^[^ ^55^ ^]^ RP‐B‐01 (SCID),^[^ ^56^ ^]^ 012T (BALB/c nude)^[^ ^57^ ^]^
**Prostate cancer**	ADENO‐CA, DC, UC, ASC, SCC,	7.3	3.8	PC‐3, DU145, LNCaP, VcaP, LAPC4, NCI‐H660 MDAPCa2a, CWR22Rv1	ZNHi001‐A ZNHi001‐B,^[^ ^58^ ^]^ IBPi002‐A,^[^ ^59^ ^]^ Pro‐iPSC	TDCM,^[^ ^60^ ^]^ BMPCa,^[^ ^61^ ^]^ ADENO‐CA,^[^ ^62^ ^]^	MSK‐PCa1‐7^[^ ^63^ ^]^ PBCre Rosa26^LSL–ERG^ mice^[^ ^64^ ^]^ hPSC‐KCs^[^ ^65^ ^]^	canine model,^[^ ^66^ ^]^ Copenhagen rat (Wistar rats^[^ ^67^ ^]^; AxC rats^[^ ^68^ ^]^; ACI/Seg rats^[^ ^69^ ^]^)	BOP (MRC rats), MNU (various rat strains), DMAB (F344/ACI rats), PhiP (F344 rats), Hormonally (Noble/Sprague–Dawley rats)^[^ ^70^ ^]^	TRAP (transgenic rats with SV40 T antigen expression),^[^ ^71^ ^]^ Crossing the Sprague‐Dawley SV40‐T rats with Lewis strain^[^ ^72^ ^]^	22RV1 (BALB/c nude),^[^ ^73^ ^]^ VcaP (CB17 SCID)^[^ ^74^ ^]^	KUCaP‐2 (Nude),^[^ ^75^ ^]^ KUCaP‐3 (SCID),^[^ ^76^ ^]^ LTL310 (NOD‐SCID)^[^ ^77^ ^]^
**Urethra cancer**	UC, ADENO‐CA, SCC, ASC,	Primary carcinomas < 1%^[^ ^78^ ^]^		HS 769.T, THUEC	UT‐iPSC^[^ ^79^ ^]^	N/A	N/A	primary urethral (61/260, 23%),^[^ ^80^ ^]^ BN rats (F: 20%, M: 6%)	BBN (SD/cShi rats),^[^ ^21^ ^]^	Expression of SV40 large T antigen^[^ ^81^ ^]^	N/A	LTL352 (NOD‐SCID)^[^ ^77^ ^]^
**Testicular cancer**	GCT (Seminoma, EC, Teratoma), non‐GCT	0.4	0.1	TCAM‐2, RT‐112, NEC8, NCC‐IT, 2102EP	SSCLCs, PGCLCs, mPGCLCs	N/A	N/A	TER/SV Mice (M: 30%), 129 Mice (M: 1%), 129/RrJ Mice (M: 5%), 129/Sv inbred strain Mice (M: 1% ‐ 5%),^[^ ^82^ ^]^ ACI rats (M: 46%)	Cadmium (rats/mice)^[^ ^83^ ^]^ PFOA^[^ ^84^ ^]^	Stra8‐Cre (Tg(Stra8‐icre)1Reb) mice conditional (floxed) allele of Pten mice, LoxP‐STOP‐LoxP‐KrasG12D transgenic mice, Oct4‐Gfp transgenic mice^[^ ^85^ ^]^	Gonadal ridges cell (129/SvJ mice)^[^ ^86^ ^]^	TC1/TC4/TC5 (NSG)^[^ ^87^ ^]^ TGCT_001/002/003 (SCID)^[^ ^88^ ^]^
**Penile cancer**	SCC (warty; papillary; basaloid; verrucous; sarcomatoid), non‐SCC (SC; MM; EMPD; ML)	0.2	0.1	149RCa, 149RM, LM156, Penl1, Penl2, PCA‐23, PCA‐5	CHOPi004‐A,^[^ ^89^ ^]^ HEL24.3,^[^ ^90^ ^]^ iPSC ‐FH2.1^[^ ^91^ ^]^	HPV18‐VIN^[^ ^92^ ^]^	N/A	EcPV‐2 related Horse model^[^ ^93^ ^]^	N/A	C57Bl/6 mice (targeted deletion of Apc/Smad4),^[^ ^94^ ^]^ FVB/N mice (targeted expression of the entire HPV16 early region)^[^ ^95^ ^]^	Penl, Penl2 (BALB/c nude)^[^ ^96^ ^]^	LTL370 (NOD‐SCID),^[^ ^77^ ^]^ HPV^−^/HPV ^+^ pSCC (NMRI‐FOXn1 mice)^[^ ^97^ ^]^

### 2.2 Bioprinted research models are advantageous

**FIGURE 2:**
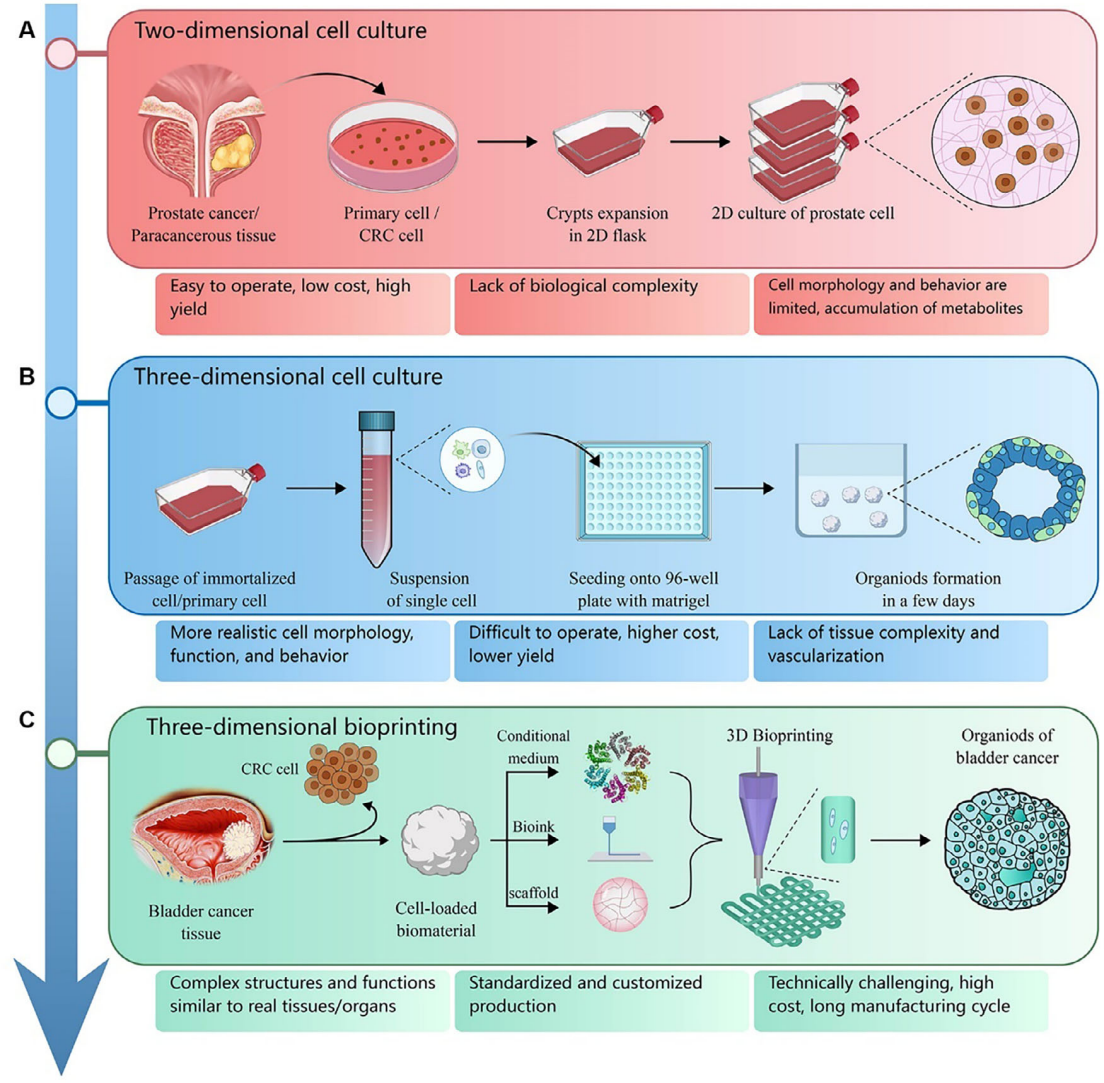
A comparison of three kinds of research models. (A) A diagram showing the experimental protocols and characteristics of two‐dimensional cell culture (2DCC). (B) A diagram of three‐dimensional cell culture (3DCC). (C) A diagram of three‐dimensional bioprinting (3DBP). These research models are arranged in chronological order of development.

Researchers are developing next‐generation technology to overcome the shortcomings of existing research models. Among them, BRMs are particularly advantageous in simulating the natural structure and characteristics of UM. This review compares three kinds of research models for graphic illustration. As shown in Figure 2A, two‐dimensional cell culture (2DCC) is most frequently used for exploring tumors. The cells are isolated from human‐derived tissue samples and then expanded and cultured in a 2D flask. 2DCC is easy to perform, with low cost and high yield. Nevertheless, it cannot mimic the biological complexity of the primary tumor and thus limits the morphology and behavior of tumor cells, hindering its application in precision medicine.^[^ ^98^ ^]^

A three‐dimensional cell culture (3DCC) model based on spheroid (co)culture has been proposed.^[^ ^99^ ^]^ As shown in Figure 2B, tumor spheroids (TS) with single or multiple cellular components can be generated by seeding the cells onto Matrigel or other superhydrophobic, nonadherent surfaces.^[^ ^100^ ^]^ For example, Olofsson et al. successfully fabricated a multicellular TS model of renal carcinoma using an ultrasound‐based culture platform.^[^ ^101^ ^]^ 3DCC exhibits more realistic cell morphology, function and behavior than 2DCC. However, its applications are still hindered by the difficulty of operation, high cost, and low yield. Moreover, 3DCC does not replicate the tissue complexity and vascularization of the primary TME.^[^ ^102^ ^]^

Bioprinting is an innovative additive manufacturing (AM) technique for the fabrication of tissue‐like constructs comprising live cells and extracellular matrix (ECM), such as bone, muscle, organ, and tumor models.^[^ ^103^ ^]^ As shown in Figure 2C, seeding cells are printed in biocompatible PBIs. The PBIs are based on natural or synthetic polymers and possess a high water content ratio, porosity, and permeability.^[^ ^12^ ^]^ Seeding cells can survive and proliferate under special cultivation conditions to obtain three‐dimensional bioprinting (3DBP) research models, especially tumor organoids. In contrast to other research models, 3DBP models that mimic the complex structures and functions of real tissues/organs can be obtained by following standardized protocols. Recently, a multimaterial bioprinting technique was developed to print multiple PBIs containing different seeding cells into one system to form more advanced heterogeneous tumor‐like constructs.^[^ ^104^ ^]^

## 3 FABRICATION OF BIOPRINTED RESEARCH MODELS

Four factors, namely, bioprinting technology, PBIs, the collection of seeding cells and the culture of a cancer model, are necessary to fabricate an ideal BRM. Researchers have done extensive work to optimize these factors. In this section, we will briefly introduce and then discuss in depth several recent advances in bioprinting technology. A summary of the other factors can be found in our previous review.^[^ ^105^ ^]^

### 3.1 Extrusion‐based bioprinting

**FIGURE 3:**
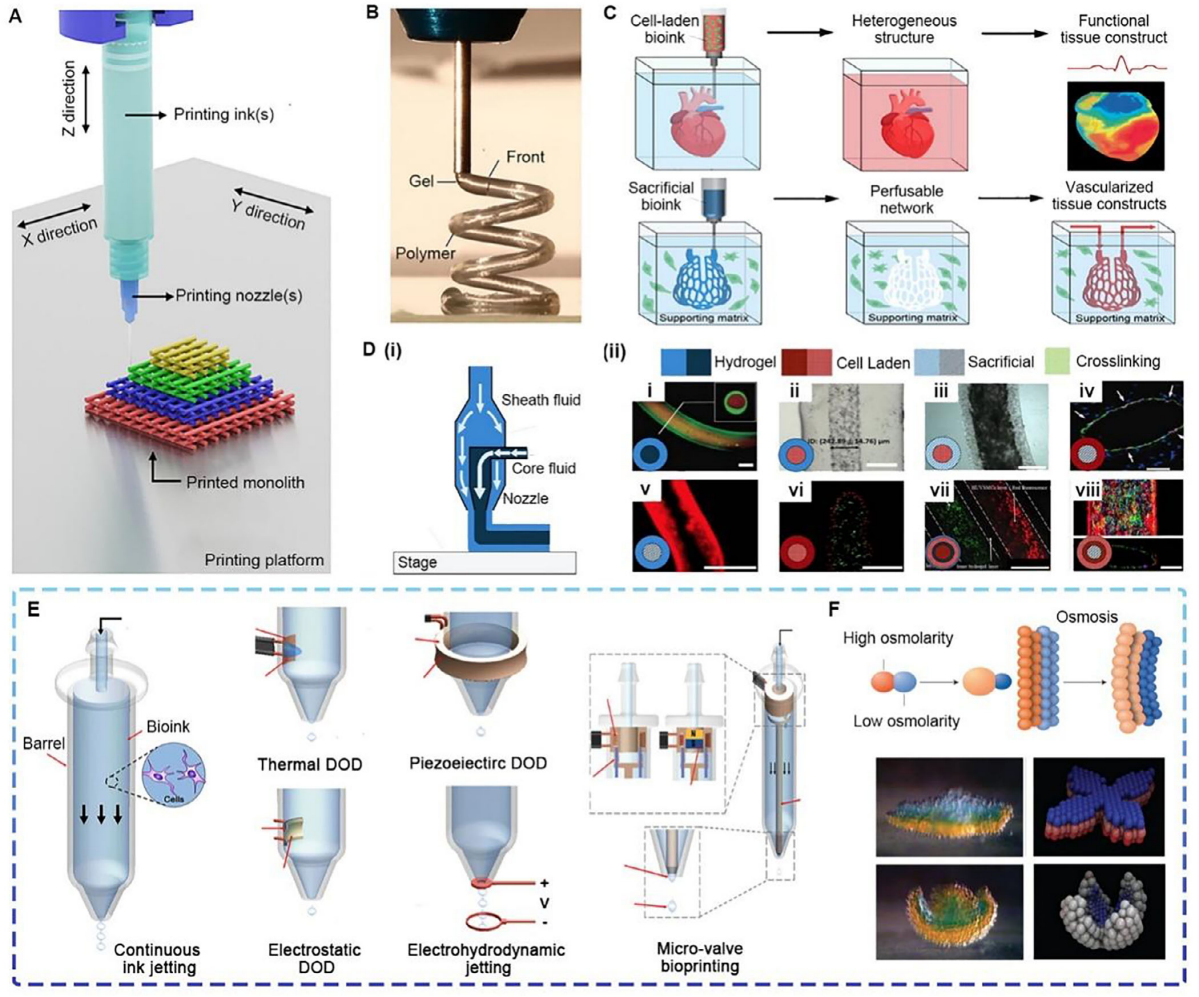
Extrusion‐based bioprinting (EBB) and droplet‐based bioprinting (DBB). (A) The setup of the EBB printer. Reproduced with permission.^[^ ^125^ ^]^ Copyright 2023, Royal Society of Chemistry. (B) 3D printed constructs prepared by EBB. Reproduced with permission.^[^ ^126^ ^]^ Copyright 2018, Springer Nature. (C) Fabrication of cell‐loaded constructs with/without vascularization by extrusion‐based embedded bioprinting (EBEB). Reproduced with permission.^[^ ^127^ ^]^ Copyright 2022, Elsevier. (D) Coaxial EBB: (i) the principle of coaxial EBB; (ii) coaxial printed constructs. Reproduced with permission^[^ ^128^ ^]^ Copyright 2021, Elsevier. (E) A diagram of DBB, including continuous ink jetting, thermal DOD bioprinting, piezoelectric DOD bioprinting, electrostatic DOD bioprinting, electrohydrodynamic jetting bioprinting, and micro‐valve (solenoid) bioprinting. Reproduced with permission.^[^ ^129^ ^]^ Copyright 2016, Biomaterials. (F) A stimuli‐responsive droplet network. Reproduced with permission.^[^ ^130^ ^]^ Copyright 2013, AAAS.

Extrusion‐based bioprinting (EBB) is a mature technology that uses a nozzle to accurately extrude PBIs for layer‐by‐layer (LBL) deposition onto substrates (Figure 3A). After printing, the PBIs immediately transform from the liquid phase to the solid phase through physical or chemical mechanisms to form the desired constructs (Figure 3B). Thermosetting polymers (TSPs), such as polylactic acid (PLA), poly‐hydroxyethyl acrylate (PHEA) and dicyclopentadiene (DCPD), are widely used due to their superior strength, rigidity and chemical stability.^[^ ^106^ ^]^ However, the biocompatibility and biodegradation of TSPs are relatively poor, limiting their applications in vivo.^[^ ^107^ ^]^ As shown in Figure 3C, EBB can fabricate cell‐loaded constructs with or without vascularization, making it suitable for simulating the TME. Seeding cells, including cell lines and primary cells from patients, are fragile in terms of viability, which places higher demands on PBIs and printing conditions. Natural PBIs, including collagen, gelatin and silk fibroin, have recently attracted increasing interest.^[^ ^108^ ^]^ Collagen has emerged as the most commonly used PBI for fabricating UM BRMs. Compared to TSPs, natural PBIs have high biocompatibility and suitable biodegradation rates but relatively poor printability and mechanical strength. Thus, EBB printing techniques for natural PBIs need further development.

Extrusion‐based embedded bioprinting (EBEB) refers to the deposition of onto a support matrix with appropriate rheological and/or mechanical properties.^[^ ^109^ ^]^ A support matrix can enable weaker PBIs to maintain the predefined pattern.^[^ ^110^ ^]^ The emergence of EBEB has significantly expanded the richness of printable PBIs and promoted the development of complex constructs in soft tissues, including biomimetic tumors and the extracellular matrix (ECM). For example, Mark et al. successfully manufactured a series of organ‐specific tissues embedded with vascular channels to exchange oxygen, nutrients, and waste.^[^ ^111^ ^]^ Vascularization is one of the fundamental characteristics of tumorigenesis.^[^ ^112^ ^]^ Coaxial bioprinting is multichannel EBB using concentric layered nozzles. As shown in Figure 3D, various high‐resolution constructs can be created by adjusting the types of PBIs within each fluid layer of the nozzle. The same nozzle size can produce different core‐sheath ratios depending on extrusion rates and the ink rheology and cross‐linking parameters.^[^ ^113^ ^]^ Moreover, straight, wavy and helical fibers can be extruded by varying the flow rate proportion of the core and sheath.^[^ ^114^ ^]^ Due to its multimaterial printing capability, coaxial printing has been used to construct highly viable glioma stem cell shell/glioma cell line core hydrogel microfibers to simulate the tumor microenvironment.^[^ ^115^ ^]^

### 3.2 Droplet‐based bioprinting

Droplet‐based bioprinting (DBB) includes inkjet and microvalve bioprinting. Inkjet bioprinting can be divided into three categories: continuous ink jetting, droplet‐on‐demand (DOD) bioprinting and electrohydrodynamic (EHD) jetting. As shown in Figure 3E, continuous ink jetting sprays the PBIs through a nozzle under pressure, and Rayleigh‐Plateau instability disperses the jet into droplets.^[^ ^116^ ^]^ DOD bioprinting uses thermal actuators (thermal DOD), piezoelectric actuators (piezoelectric DOD) or electrostatic forces (electrostatic DOD) to generate droplets.^[^ ^117^ ^]^ Compared to continuous ink jetting, DOD bioprinting generates droplets only when needed, making them more economical and controllable for fabricating BRMs.^[^ ^118^ ^]^ However, pushing cell‐laden droplets through small nozzles requires high pressure and can harm the seeding cells. To solve this problem, EHD jetting was developed to use an electric field between the printing head and substrate to pull PBI droplets over the printing head hole, eliminating the need for high pressure.^[^ ^119^ ^]^ Thus, EHD jetting is more suitable for bioprinting BRMs that require minimal nozzle diameters (≤100 μm) and highly concentrated PBIs (>20% w/v).^[^ ^120^ ^]^

Microvalve bioprinting can dispense cell‐laden droplets by using electromechanical valves. Compared to piezoelectric DOD bioprinters, microvalve bioprinters require a lower range of pneumatic pressure, making them less likely to damage the viability of seeding cells.^[^ ^121^ ^]^ However, the microvalve bioprinter produces larger droplets than other DBB modes when using the same size nozzle, resulting in less ideal spatial resolution.^[^ ^122^ ^]^ Only by improving spatial resolution can more complex constructs be printed in limited space, thereby better simulating the characteristics of the TME. Recently, the concept of 4D bioprinting has been proposed to fabricate stimuli‐responsive mimics of live tissue.^[^ ^123^ ^]^ Herein, it is noted that EBB can integrate with 4D bioprinting to prepare next‐generation BRMs that self‐deform and self‐move under physiological conditions or mimic the communication and cooperation between living cells. For example, Gabriel et al. reported a droplet network consisting of tens of thousands of high osmotic pressure‐responsive and low osmotic pressure‐responsive droplets (Figure 3F). The network allows rapid electrical communication along a specific path and can be programmed into designed structures by osmolarity gradients. Based on the above structure, the constructs can be extensively used in cancer models, soft robots, drug delivery systems and diagnostic tools.^[^ ^124^ ^]^

### 3.3 Laser direct‐write bioprinting

**FIGURE 4:**
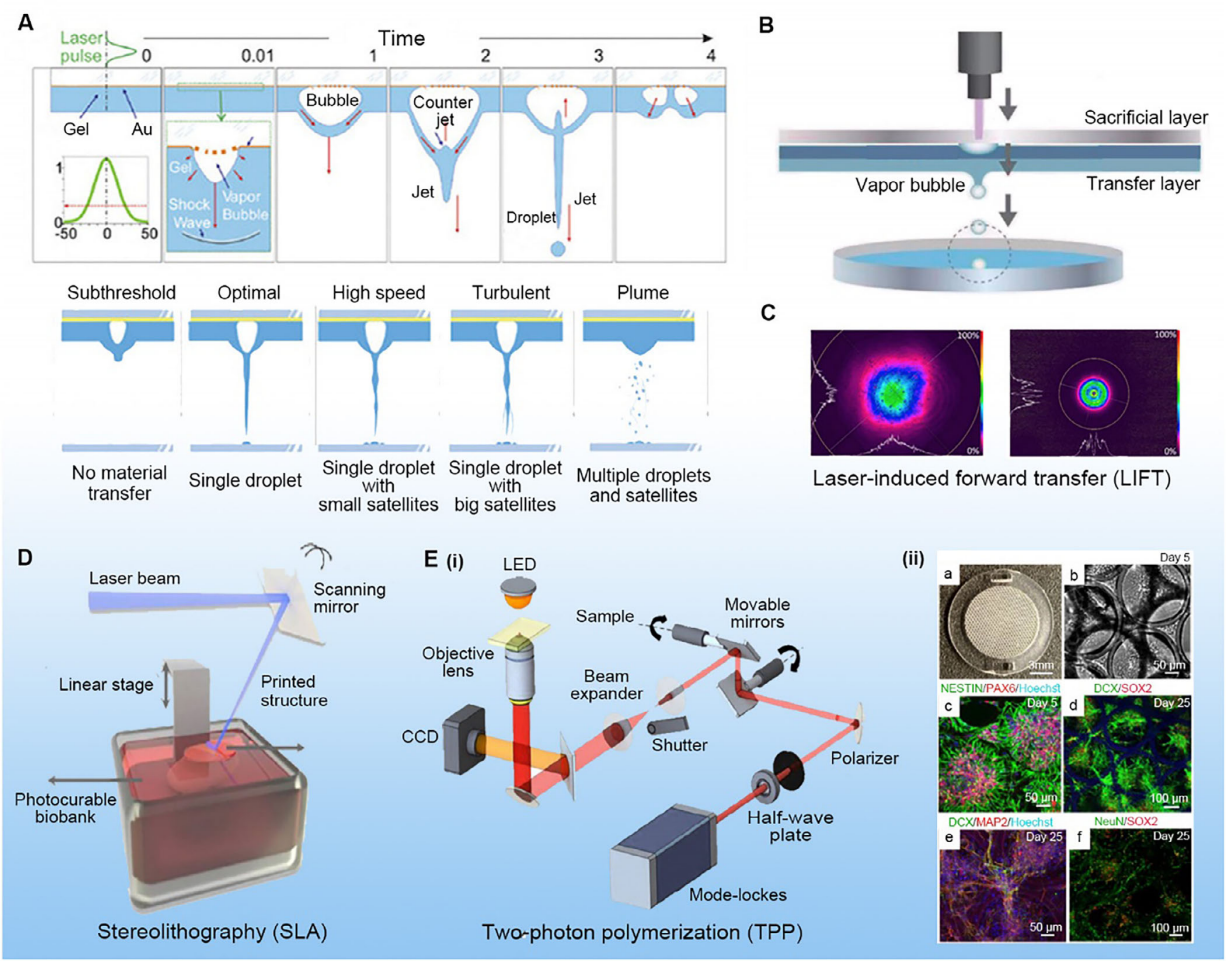
Laser direct write (LDW) bioprinting. (A) The formation process during LDW bioprinting; Red arrows indicate the direction of fluid movement. Reproduced with permission.^[^ ^141^ ^]^ Copyright 2020, WHOICE. (B) A schematic showing the laser‐induced forward transfer (LIFT). Reproduced with permission.^[^ ^142^ ^]^ Copyright 2019, Elsevier. (C) Beam profiler images of laser pulses used for the blister‐actuated LIFT bioprinting. Reproduced with permission.^[^ ^143^ ^]^ Copyright 2019, MDPI. (D) A schematic overview of the stereolithography. Reproduced with permission.^[^ ^144^ ^]^ Copyright 2022, Elsevier. (E) A schematic overview of two‐photon polymerization (TPP): (i) the components of the TPP system; (ii) generation of a 3D neuronal cell culture system. Reproduced with permission.^[^ ^145^ ^]^ Copyright 2021, MDPI.

Laser direct writing (LDW) bioprinting is a nozzle‐free forward transfer technique that uses pulsed lasers to deposit PBIs from a “printing ribbon” onto a “receiving substrate.” Compared to EBB and DBB, LDW has a relatively mild impact on cell viability since it does not produce high temperature or shear stress. Previous work demonstrated that the cell viability of LDW can reach 85%−95%, fulfilling the needs of BRMs.^[^ ^131^ ^]^ Meanwhile, LDW allows the integration of various types of seeding cells and cytokines into one platform to recapitulate the heterogeneity and composition of living tissues and tumors.^[^ ^132^ ^]^ Usually, LDW is divided into selective laser‐induced forward transfer (LIFT), stereolithography (SLA), and two‐photon polymerization (TPP) technologies.^[^ ^133^ ^]^ In LIFT, local heating occurs between the laser pulse and the absorption layer to produce explosive boiling of water or gel. High‐pressure bubbles form various types of jets or cause the separation of one or more droplets (Figure 4A). As a result, the PBIs are transferred from the donor matrix to the receptor matrix (Figure 4B).^[^ ^134^ ^]^ The size and shape of the sprayed PBIs are mainly influenced by the diameter and profile of the incident laser beam (Figure 4C). Researchers have developed a series of modified LIFT processes, including bubble‐assisted LIFT (BA‐LIFT), matrix‐assisted pulsed laser evaporation (MAPLE) + LIFT, thermal imaging (TI) and laser‐induced thermal imaging (LITI), improving their effectiveness in fabricating advanced BRMs.^[^ ^135^ ^]^

In SLA, a laser beam is used for the LBL irradiation of a photosensitive PNI through a galvanometer scanner and optical devices to prepare 3D constructs (Figure 4D).^[^ ^136^ ^]^ It allows the large‐scale manufacture of 3D constructs from the micrometer scale to macroscopic structures and is thus adaptable to a series of applications, including biodegradable scaffolds for TS and organoids, a matrix to simulate the TME, and a microfluidic chip for drug discovery.^[^ ^137^ ^]^ In TPP, an optical objective lens is used to focus a near‐infrared femtosecond laser with a wavelength of 800 nm to generate a nonlinear visual effect, causing the monomer solution to reach an excited state and trigger polymerization after absorbing two photons (Figure 4Ei).^[^ ^138^ ^]^ For TPP, the interaction between the laser and PBIs is not limited to the photoresist surface, allowing the fabrication of multiscale constructs with arbitrary micro‐nano geometric shapes and subdiffraction features.^[^ ^139^ ^]^ Therefore, TPP is widely used to fabricate bioactive 3D devices, including controllable 3D culture systems that mimic the characteristics of cell invasion and proliferation (Figure 4Eii).^[^ ^140^ ^]^

### 3.4 Projection‐based 3D bioprinting

**FIGURE 5:**
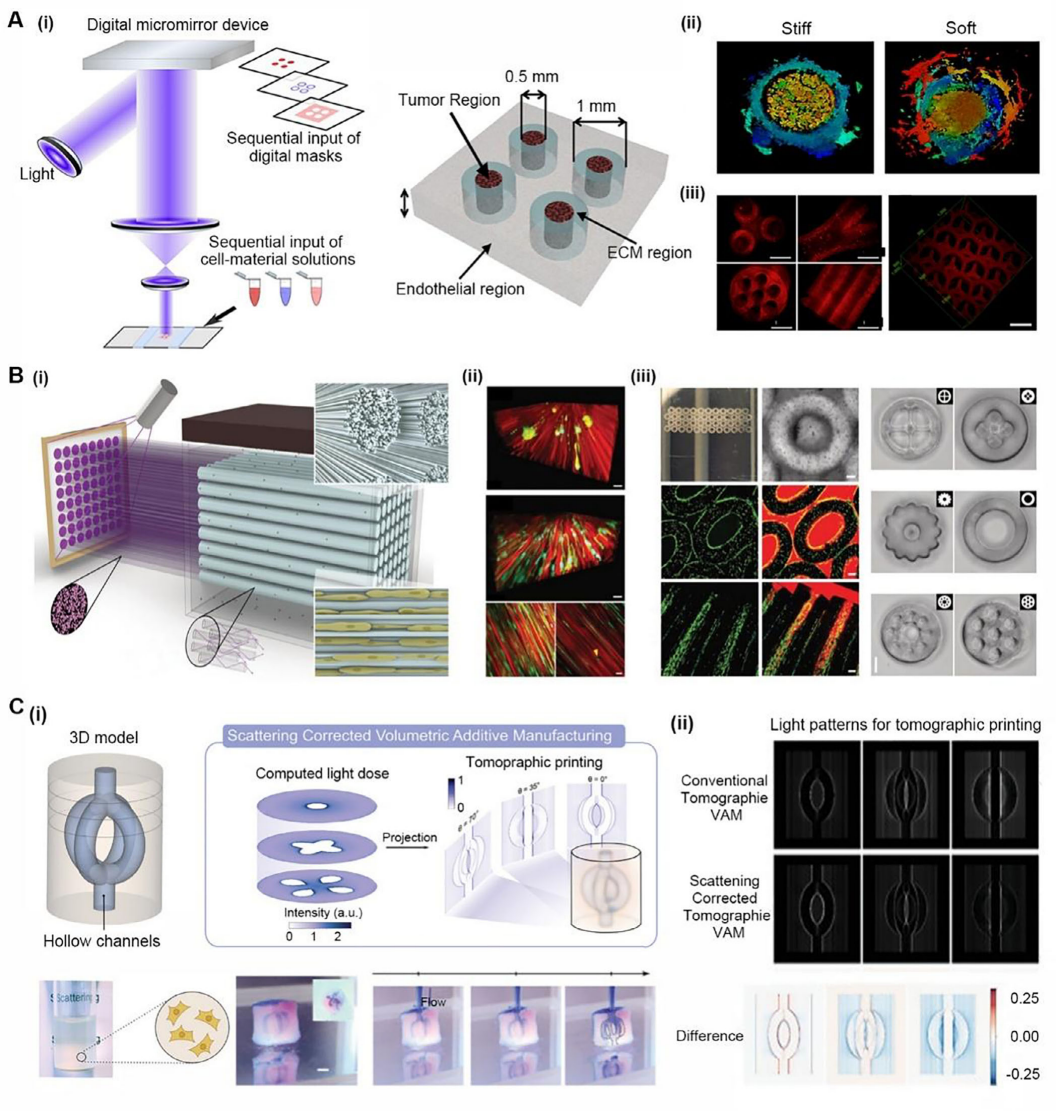
Projection‐based 3D bioprinting. (A) A multi‐step digital light processing‐based (MSDLP) bioprinting was used to prepare research models with regionally varied biophysical properties: (i) the setup of MSDLP bioprinting and the model dimensions; (ii) invasion patterns of tumor cells in models with varied stiffness; (iii) complex 3D structures fabricated by MSDLP bioprinting. Reproduced with permission.^[^ ^155^ ^]^ Copyright 2021, Wiley‐VCH. Reproduced with permission.^[^ ^156^ ^]^ Copyright 2016, Elsevier. (B) A filamented light (FLight) strategy to prepare hydrogels containing highly aligned microfilaments with efficient cell guidance properties: (i) a diagram showing the principle of FLight; (ii) fluorescence images of cell‐laden hydrogels; (iii) images of complex hydrogels prepared by FLight/multi‐FLight. Reproduced with permission.^[^ ^157^ ^]^ Copyright 2022, Wiley‐VCH. (C) Scattering‐corrected volumetric additive manufacturing (VAM) could print complex geometries with hollow channels: (i) a diagram showing the principle of scattering‐corrected VAM; (ii) light patterns projected at different angles with and without correction. The difference shows by where and how much the correction is applied to account for scattering. Reproduced with permission.^[^ ^158^ ^]^ Copyright 2022, Wiley‐VCH.

As shown in Figure 5Ai, projection‐based 3D bioprinting (PBP) consists of a platform, ink cartridge and projector. Before PBP, the digital model of the desired construct is sliced by software, imported into a digital micromirror device chip, and then reflected and projected onto an ink cartridge. The exposed PBI undergoes chemical crosslinking, while the unexposed ink remains liquid.^[^ ^146^ ^]^ PBI and photoinitiators with high photopolymerization rates are needed. Moreover, light absorbers are widely used to adjust the depth of light penetration, improving the spatial resolution of PBPs.^[^ ^147^ ^]^ As shown in Figure 5Aii,iii, various 3D constructs, such as tumor invasion models, vascularized networks and hollow conduits, have been developed.^[^ ^148^ ^]^ Gou et al. found that PBP could fabricate a liver lobule microstructure BRM from PBIs with integrated functional nanoparticles.^[^ ^149^ ^]^

Filament light (FLight) based on PBP is a novel biomanufacturing technique that can quickly fabricate centimeter‐scale hydrogels composed of unidirectional microfilament constructs within seconds (Figure 5Bi). Traditional hydrogels hinder the diffusion of oxygen and nutrients.^[^ ^150^ ^]^ The microchannels of hydrogels prepared by FLight have an ultrahigh aspect ratio (>700:1) and thus have superior cytocompatibility and are effective in arranging cells into neat tissues (Figure 5Bii). The microchannels can also support the survival and proliferation of seeding cells inside the hydrogels, which is vital for cell‐laden BRMs. As shown in Figure 5Biii, multi‐FLight has been developed to fabricate hydrogels with more complex structures. Multidirectional microchannels can be prepared within a single PBI by changing the direction of the projection. Moreover, multimaterial/cell constructs can be achieved by sequentially exchanging the types of PBIs with or without cells.

Volume bioprinting (VBP) is a revolutionary technology.^[^ ^151^ ^]^ It utilizes volume imaging modes, including computer tomography, to induce the cross‐linking of PBIs through multiangle projection‐based light accumulation.^[^ ^152^ ^]^ VBP breaks through the geometric limitations of LBL additive manufacturing techniques and exhibits good spatial accuracy and printing speed. The resolution of printed matter is greatly influenced by the properties of PBIs and the potential optical aberrations inherent in the experimental setup.^[^ ^153^ ^]^ Light scattering caused by opaque materials can disrupt the as‐designed spatial information.^[^ ^154^ ^]^ As shown in Figure 5C, Jorge et al. proposed a scattering‐corrected volumetric additive manufacturing (VAM) technique to solve this problem. Scattering‐corrected VAM is especially suitable for printing turbid BRMs using cell‐laden PBIs.

### 3.5 Magnetic 3D bioprinting

**FIGURE 6:**
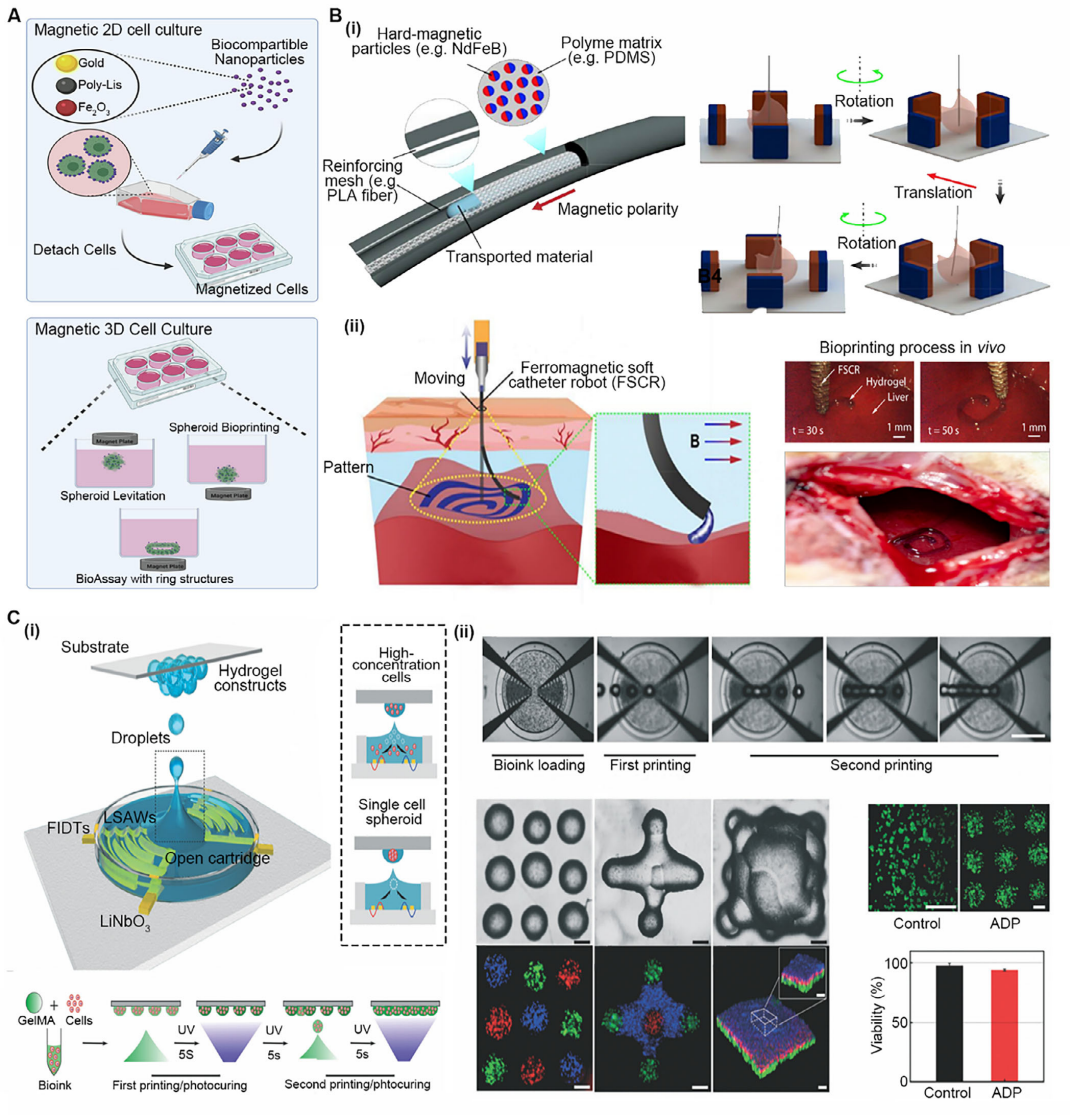
Magnetic 3D (M3D) bioprinting and acoustic droplet (ADP) bioprinting. (A) A comparison of magnetic 2D and 3D cell culture. Reproduced with permission.^[^ ^168^ ^]^ Copyright 2021, Elsevier. (B) M3D bioprinting was preliminarily used in vivo: (i) a diagram showing ferromagnetic soft catheter robot (FSCR) that is composed of soft polymer matrix with dispersed hard‐magnetic particles and polylactide reinforcing mesh. The movement of FSCR could be maneuvered by the rotation and translation of four permanent magnets; (ii) minimally invasive M3D bioprinting with functional bioinks inside the human body. Reproduced with permission.^[^ ^169^ ^]^ Copyright 2021, Springer Nature. (C) ADP bioprinting was developed: (i) the ejected droplets containing suspended cells at a high concentration or single cell spheroids deposit on a receiving substrate to form 3D hydrogel constructs; ii) the images of heterogeneous, complex, and diverse hydrogel constructs with high cell viability are presented. Reproduced with permission.^[^ ^166^ ^]^ Copyright 2021, Royal Society of Chemistry.

Magnetic 3D bioprinting (M3D) can be used to fabricate BRMs for magnetic cell culture (Figure 6A). For 2DCC, the cells are first incubated with magnetic nanoparticles (MNPs) in a two‐dimensional device. These MNPs bind to cells through nonspecific interactions. Subsequently, the magnetized cells are dissociated and exposed to a magnetic field generated by neodymium magnets to induce aggregation into a 3D construct. For 3DCC, three types of M3D have been reported, including spheroid levitation, spheroid bioprinting, and BioAssay with ring structures. For spheroid levitation, a neodymium magnet is placed on top of the culture plate to facilitate the aggregation of magnetized cells at the liquid–air interface, forming a levitated construct through cell‒cell and cell–ECM interactions.^[^ ^159^ ^]^ Spheroid bioprinting involves seeding magnetized cells on a plate with independent magnet devices, applying a mild magnetic force to induce cell aggregation and matrix formation, and printing spheres at the bottom of each well.^[^ ^160^ ^]^ BioAssay with ring structures involves suspending and culturing magnetized cells with ECM to form aggregates, enzymatically dissociating these aggregates into dispersed cells, and then transferring the culture plate onto circular neodymium magnets to induce circular cell aggregation.^[^ ^161^ ^]^

M3D provides a standardized protocol that allows the controllable movement and aggregation of magnetized UM cells and the consistent fabrication of UM TSs. In recent years, M3D has been used to generate various scaffold‐free constructs, such as human fetal osteoblast (hFOB) spheroids as a model of bone tissue engineering and glioblastoma cell (U87) spheroids as a model of wound healing.^[^ ^162^ ^]^ In addition to generating homotypic cultures (one cell source), M3D can produce heterotypic cultures (multiple cell sources) and organotypic multicellular spheres. For example, Caroline et al. developed a heterotypic M3D culture composed of human ovarian cancer cells (CAISMOV24) and peripheral blood mononuclear cells (PBMCs) to recapitulate the TME of ovarian cancer.^[^ ^163^ ^]^

M3D can be applied in vivo in combination with minimally invasive bioprinting devices. For example, Zhou et al. developed a ferromagnetic soft catheter robot (FSCR) system. As shown in Figure 6Bi, it is designed as a slim rod‐shaped structure containing dispersed hard magnetic microparticles (neodymium iron boron, NdFeB), which can be guided to internal regions of the body by remote magnetic actuation. Compared to conventional bioprinting systems using rigid nozzles, the FSCR features a magnetoactive soft nozzle that can print in situ over a large area through a small incision (Figure 6Bii). The FSCR can print different patterns using multiple PBIs, such as lesion healing creams, silicones, silver pastes, and electrode gels on both flat and curved surfaces. In recent years, remote intelligent applications of surgical DaVinci Robotics have attracted increasing interest. This digital strategy of FSCR driven by a magnetic field, without the process of PBI magnetization, is emerging as a novel prospect for the translation of the M3D technique from bench to bedside.

### 3.6 Acoustic droplet bioprinting

Acoustic droplet bioprinting (ADP) utilizes focused sound waves to eject single droplets at the liquid‐air interface without a nozzle (Figure 6Ci).^[^ ^164^ ^]^ In this way, high cell concentrations (>10^8^ cells/mL) and even single‐cell spheroids can be reliably printed without clogging and with high cell viability (>94%). The ejected droplets can be deposited on a receiving substrate in a noncontact manner and accurately arranged into various 2D patterns. These droplets can be further stacked in three dimensions using an LBL deposition approach.^[^ ^165^ ^]^ ADP has been widely used to fabricate TSs and organoids. To achieve high‐fidelity ADP, a strategy of two‐step printing has been proposed. The water droplets generated by the first printing act as fixed fences to effectively mitigate droplet flow, resulting in hydrogel layers with good shape reproducibility. Furthermore, with the use of three acoustic printers that can store different PBIs, droplets can be printed on demand. Complex, heterogeneous, and reproducible point arrays (1D), crossroads (2D), and pyramids (3D) can be accurately constructed (Figure 6Cii). Through the ADP method, Chen et al. printed a tumor spheroid (CAL27)–CAF‐cocultured microdevice that can simulate tumor invasion in vivo.^[^ ^166^ ^]^ Gong et al. prepared bladder cancer organoids within one week and provided variability predictions for immunotherapy in individual patients.^[^ ^167^ ^]^

## 4 APPLICATIONS OF BIOPRINTED RESEARCH MODELS

Through fabricating advanced research models, researchers will produce a deeper understanding of UM. BRMs with preprogrammed structures and functions have multiple advantages over conventional research models and are thus successfully applied in a wide range of fundamental research. In recent years, a series of milestone achievements has been made in this field. To give an up‐to‐date summary of the research advances, this section is divided into 6 parts, each focusing on a specific application. An in‐depth discussion of each application is provided along with a forward‐looking perspective.

### 4.1 Tumor spheroids and organoids

**FIGURE 7:**
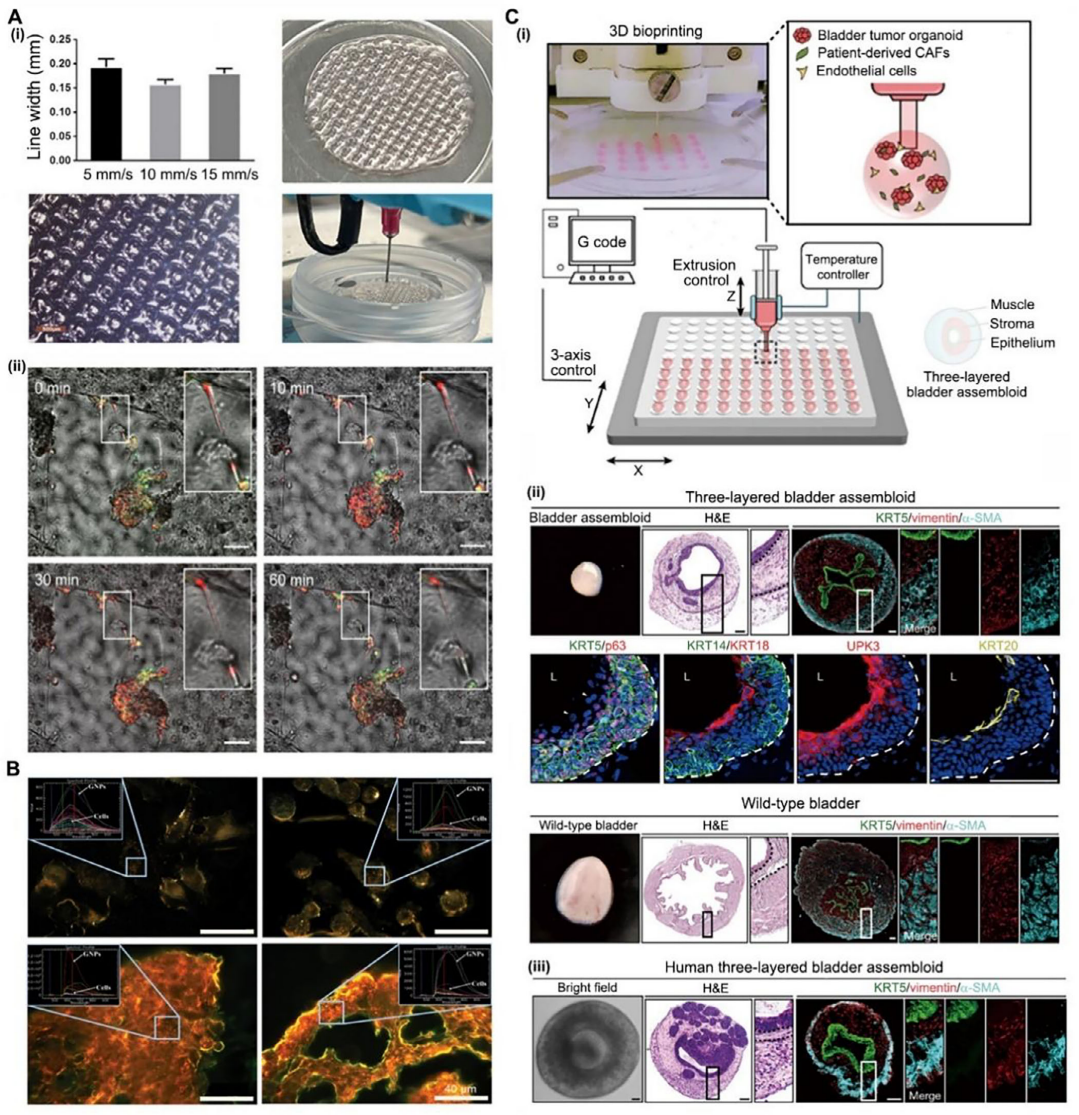
Tumor spheroids and organoids. (A) Spheroids for investigating TNT tumor structures: (i) bioprinted scaffolds were prepared as carriers of multicellular spheroids; (ii) in situ observation of mitochondria being trafficked through TNT‐like projections. Scale bar: 100 μm. Reproduced with permission.^[^ ^182^ ^]^ Copyright 2021, Elsevier. (B) Spheroids for predicting the response to targeted therapy. Reproduced with permission.^[^ ^183^ ^]^ Copyright 2022, Springer Nature. (C) Assembloids for modeling mature organ architecture and associated tissue microenvironments of bladder: (i) the reconstitution process of bladder assembloids; (ii) assembloids comprising three distinct compartments, organized tightly to form the structures similar to that of the wild‐type bladder; (iii) patient‐derived bladder assembloids. Reproduced with permission.^[^ ^184^ ^]^ Copyright 2020, Springer Nature.

Bioprinting allows the layering and positioning of cells into 3D constructs to establish a nano‐microenvironment with a programmed spatial arrangement of different cells. These cell assemblies can enable the analysis of molecular diffusion and the observation of cell behavior and communication.^[^ ^170^ ^]^ For example, Helena et al. reported a bioprinted hydrogel platform for 3D cell culture and study of the tunneling nanotube (TNT)‐like structures of renal cancer (Figure 7A). TNTs are thin membrane tubes that connect to distant cells and serve as intercellular transport channels for various cargoes.^[^ ^171^ ^]^ Renal cancer cells can self‐assemble in this platform, form TNT‐like protrusions, and transport mitochondria between adjacent cells. This BHP was controllable and reproducible, providing an ideal experimental microenvironment for investigating the relevance of TNTs to both tumorigenesis and antitumor drug susceptibility.

Tumor spheroids (TSs) refer to the 3D culture of a single cell line with the transient assembly of cell organization. TSs prepared by bioprinting can capture important details, such as the packaging densities of different cells and the concentration‐dependent effect of the ECM, which are not found in conventional 2DCC. Bioprinted TSs are superior for recapitulating the therapeutic response in vitro.^[^ ^172^ ^]^ For example. Bromma et al. fabricated a TS of prostate cancer to test combination therapy with gold nanoparticles (GNPs) and docetaxel. GNPs are taken up by targeted cells mainly through receptor‐mediated endocytosis.^[^ ^173^ ^]^ After injection in vivo, GNPs can move throughout the body and nonspecifically interact with surrounding tissues under the regulation of the TME and immune system.^[^ ^174^ ^]^ TSs can imitate the complexity of tumors more accurately than cell lines. Smaller GNPs can penetrate into deeper layers of TSs and be internalized into targeted cells that are difficult to reach (Figure 7B).

Organoids refer to the 3D culturing of multiple cell lineages derived from stem cells, which recapitulate the physiological parameters of organs. Organoids can be cultured in vitro for a longer time than TSs. Organoids can model one or more components of normal tissues, such as the mucosal and smooth muscle layers of the bladder.^[^ ^175^ ^]^ For example, Kim et al. bioprinted a series of bladder “assembloids” using two tissue matrix components, fibroblasts (mouse embryonic fibroblasts), endothelial cells (HULECs), and muscle layers (Figure 7C). Based on these assembloids (organoids), researchers found that the inhibition of matrix hedgehog signaling reduced the proliferation of epithelial and matrix cells. Bioprinted organoids (regardless of subtype) exhibit lower therapeutic responses to chemotherapy drugs than 2D culture.^[^ ^176^ ^]^ This reveals the poor delivery of chemotherapy drugs to tumor sites because the stroma of solid tumors is denser than that of normal tissue.

Bioprinted TSs and organoids can be transplanted into immune‐deficient animals to fabricate humanized research models in vivo.^[^ ^177^ ^]^ The advantages and disadvantages of a series of humanized research models were summarized and analyzed in previous reviews.^[^ ^178^ ^]^ Notably, such models were frequently compared with a patient‐derived xenograft (PDX) model, which is created by transplanting fresh tumor tissue into immune‐deficient animals and allowing the tumor tissue to grow into neotumors.^[^ ^179^ ^]^ Worldwide, fresh tumor tissue from patients is rare and invaluable for fundamental research, greatly limiting the development of PDX models. This issue can be well solved by bioprinted TSs and organoids, as they can be amplified in programmed culture conditions before in vivo transplantation.^[^ ^180^ ^]^ Unfortunately, the fabrication and culture of bioprinted TSs and organoids are time‐consuming and expensive, and the success rate is low. Their ability to model the characteristics of primary tumors is not as good as that of PDX.^[^ ^181^ ^]^

### 4.2 Biomimetic tumor microenvironment

The tumor microenvironment (TME) is a bidirectional, dynamic and complex interaction network among cancer cells, tumor vascular systems, matrix components (such as fibroblasts), and host immune cells that support tumor development.^[^ ^185^ ^]^ The TME includes varied cell populations, including cancer‐associated fibroblasts (CAFs) and tumor‐associated macrophages (TAMs), in different tumor stages and coordinates with the immune system to regulate the biological behavior of cancer cells.^[^ ^186^ ^]^ In recent years, bioprinting technology has been used to fabricate biomimetic research models of the TME in vitro. Several representative advances in this subfield are highlighted below.

**FIGURE 8:**
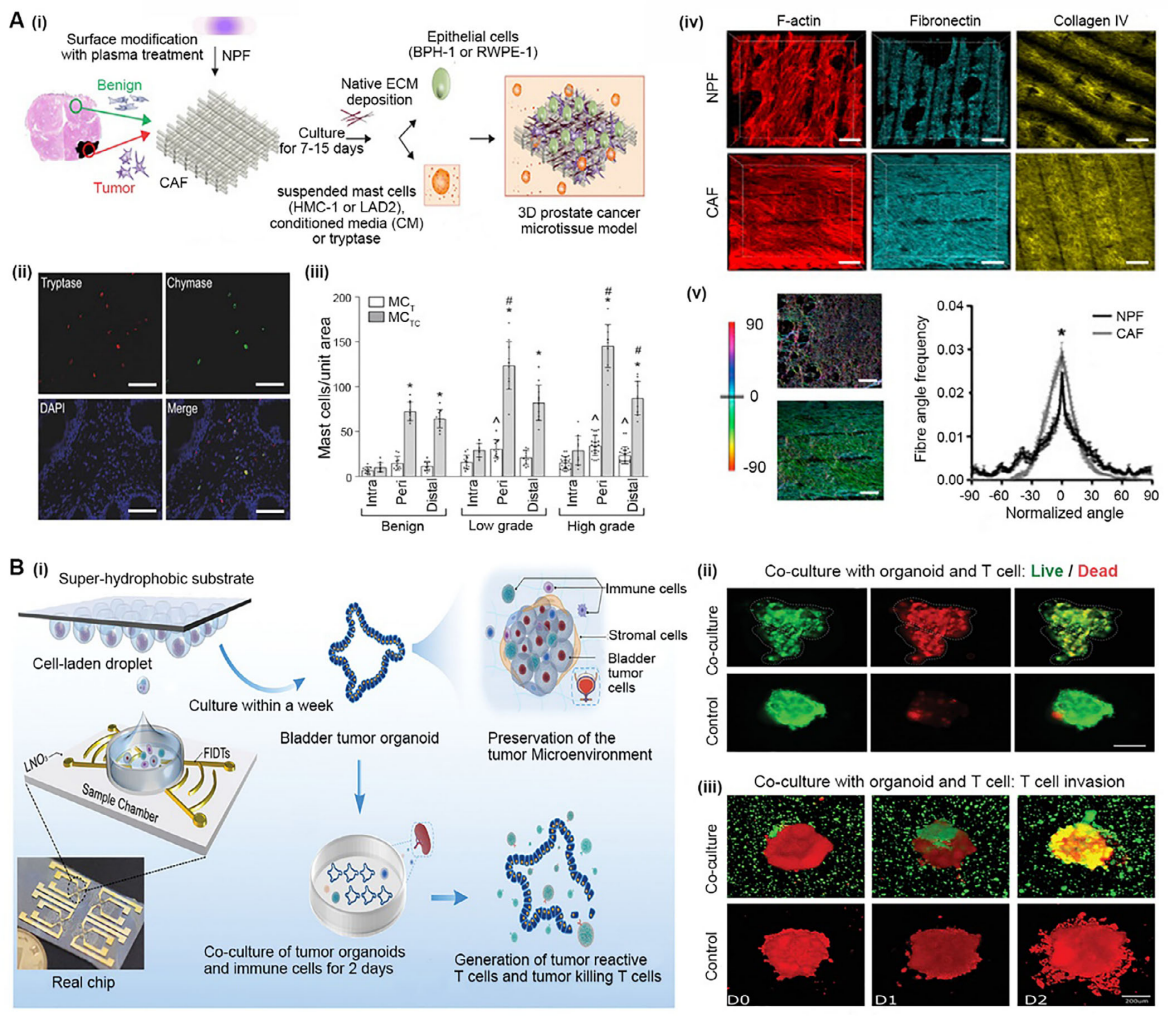
Biomimetic tumor microenvironment. (A) A tissue engineered model for modeling the TME of human PCa: (i) a schematic showing the preparation process of the model; (ii,iii) tryptase‐positive mast cells are the dominant sub‐population of mast cells in PCa; (iv,v) 3D microtissues were obtained by culturing non‐malignant prostatic fibroblasts (NPFs) and cancer‐associated fibroblasts (CAFs) in bioprinted poly‐caprolactone scaffolds. Reproduced with permission.^[^ ^193^ ^]^ Copyright 2019, Elsevier. (B) A organoids‐based model for modeling the immune TME of BCa: (i) fabrication of the model using acoustic droplet bioprinting; (ii,iii) Co‐culturing with organoids induced tumor reactivity of autologous T cells with high cell viability. Reproduced with permission.^[^ ^167^ ^]^ Copyright 2021, Wiley‐VCH.

Recently, a cell‐laden BRM of PCa has been reported for investigation of the crosstalk between CAFs and mast cells. CAFs can be activated by cancer cells to produce a large number of cancer‐associated cytokines and chemokines.^[^ ^187^ ^]^ CAFs can also reshape the ECM by secreting matrix metalloproteinases (MMPs) to form linear tracks to facilitate tumor invasion.^[^ ^188^ ^]^ Mast cells can expand in the early stages of malignant tumors and enhance the morphometric transition of benign epithelia via a tryptase‐mediated mechanism.^[^ ^189^ ^]^ As shown in Figure 8Ai, Pereira et al. seeded primary patient‐derived fibroblasts from matched nonmalignant (NPF) and malignant (CAF) prostates into a bioprinted polycaprolactone (PCL) scaffold and cultured it for 7–15 days to form a microtissue construct. The construct was then cocultured with mast cells and conditioned medium containing tryptase. The results showed that tryptase‐positive mast cells (MC_TC_) were the predominant subpopulation of mast cells in PCa (Figure 8Aii,iii), and the mast cells could effectively alter the CAF‐induced morphological transformation of benign epithelial cells and collagen deposition compared to NPF (Figure 8Aiv,v). Using this cell‐laden BRM, researchers successfully confirmed the direct relationship between CAFs and mast cells in PCa.

The interplay of different cells in the TME can also be investigated using organoid‐based BRMs. Existing research models are insufficient for such applications. For example, a coculture model based on classic 2DCC cannot fully reproduce the dynamic characteristics and complexity of the TME.^[^ ^190^ ^]^ Bioprinted organoid models can generate intricate structures composed of multiple cell types and biomaterials used as ECM analogs and thus are superior to coculture models ^[^ ^191^ ^].^ However, it usually takes a long time to prepare immune‐related organoids using clinical samples.^[^ ^192^ ^]^ Faced with these challenges, Gong et al. developed ADP technology to prepare bladder cancer organoids with controllable size in one week (Figure 8Bi). The organoids not only mimic the structural characteristics of bladder cancer but also retain an immune response ability similar to that of parental tissues. As shown in Figure 8Bii, autologous T cells could be cocultured with the organoids with good cell viability. As shown in Figure 8Biii, autologous T cells were induced to differentiate into tumor‐reactive T cells and tumor‐killing T cells and invaded the interior of organoids in response to immune stimulation. The results from these organoid‐based BRMs were highly consistent with the real events that occur in vivo. Notably, research on the TME is of increasing interest. It is speculated that the types and applications of organoid‐based BRMs of UM will be greatly enriched in the near future.

### 4.3 Models of cancer metastasis

**FIGURE 9:**
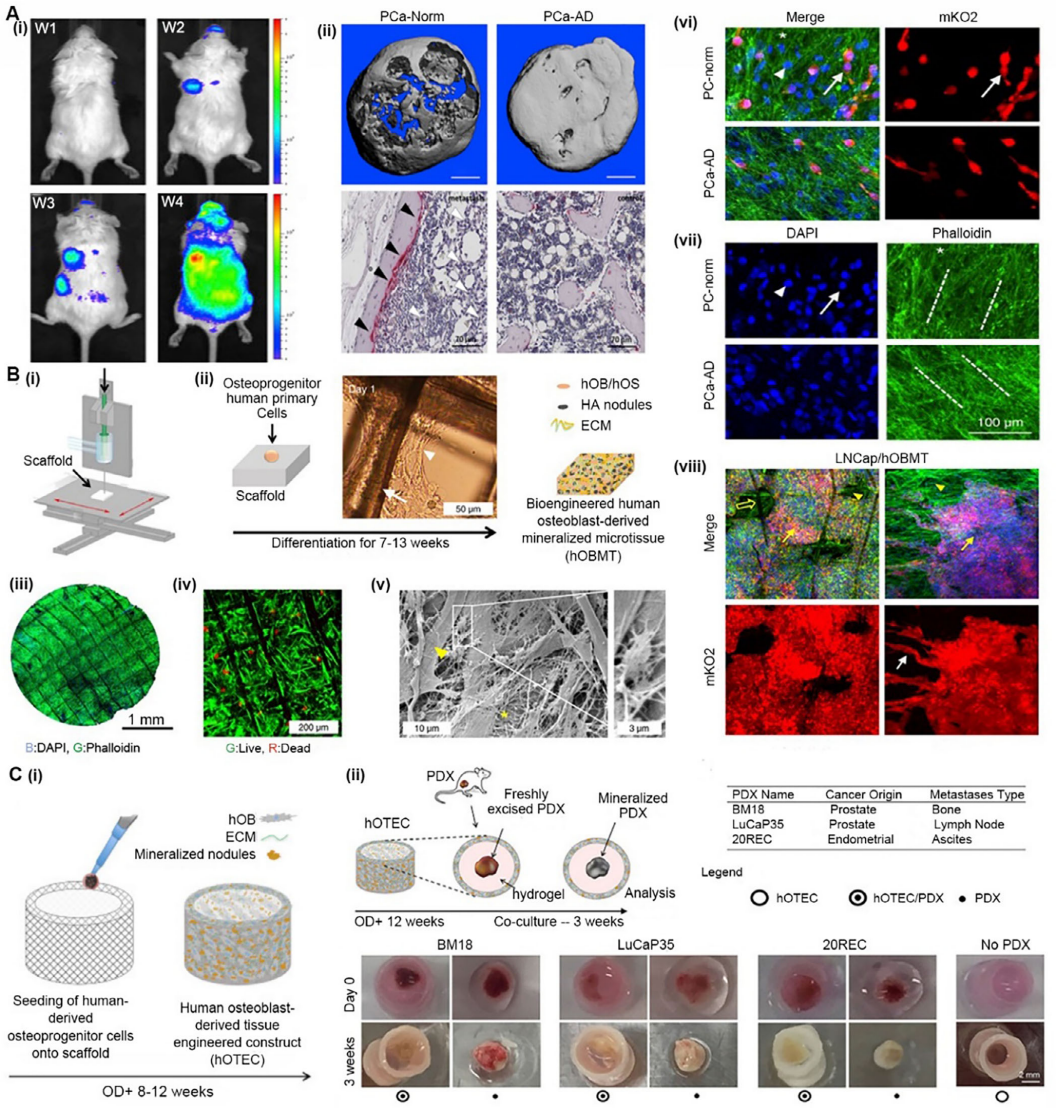
Models of cancer metastasis. (A) A tissue engineering bone model (hTEBCs) of prostate cancer metastasis in vivo: (i) the hTEBCs served as a homing site for human prostate cancer (PC3) cells; (ii) PC3 cells recapitulated their typical osteolytic metastatic growth pattern in hTEBCs. Reproduced with permission.^[^ ^196^ ^]^ Copyright 2014, Elsevier. (B) An osteoblastic metastasis model (hOBMT) for delineating the response of PCa to ADT: (i) fabrication of scaffold by melt electrowriting (thermal EBB); (ii) co‐culturing of human primary osteoprogenitor cells with the scaffold; (iii,iv) the obtained hOBMT showed high cellular organization, strong directional actin filaments and more than 80% cell viability; (v) the hOBMT showed dense ECM deposition (asterisk), osteoblastic cells (arrow head), and osteocytic cells (inset); (vi,vii) co‐culturing of hOBMT and LNCaP cells; (viii) LNCaP cells aggregated in response to androgen deprivation in the hOBMT model. Reproduced with permission.^[^ ^199^ ^]^ Copyright 2019, Springer Nature. (C) An osteoblastic metastasis model (hOTECs) for revealing the osteomimicry of PCa in vitro: (i) a schematic showing the fabrication process of hOTECs; (ii) establishing the model by combining the PDX and hOTECs. Reproduced with permission.^[^ ^200^ ^]^ Copyright 2019, Elsevier.

Tumor cells can metastasize to various tissues and organs, such as bone, lung, liver, peritoneum, and brain tissue. PCa is the most common metastatic tumor among UMs, with approximately 60–80% of late‐stage PCa patients experiencing bone metastasis.^[^ ^194^ ^]^ The metastasis of PCa is divided into four overlapping phases: colonization, dormancy, reactivation, and rebuilding. The interplay between bone cells and PCa cells has a dominant role.^[^ ^195^ ^]^ Currently, researchers are attempting to investigate this metastasis process in vivo using a series of BRMs of PCa. For example, Holzapfel et al. designed a morphologically and functionally intact BRM to study the homing behavior of PCa cells.^[^ ^196^ ^]^ Briefly, human mesenchymal progenitor cells (hMPCs) were first seeded onto a biodegradable tubular scaffold and then subcutaneously transplanted into immunodeficient mice for 14 weeks, allowing the formation of mineralized human tissue‐engineered bone constructs (hTEBCs). As shown in Figure 9A, the hTEBCs could serve as a target site of PC3 cells implanted via the left ventricle. Furthermore, metastatic cells could proliferate within hTEBCs with their typical growth pattern, leading to extensive bone loss and destruction. Compared to conventional bone metastasis based on tumor cell injection, the model of hTEBCs is of great importance in improving the success rate of modeling and expanding the potential for revealing the dynamic metastasis process of UM.

BRMs could be used to predict the drug response of metastatic UM in vitro. For example, androgen deprivation therapy (ADT) has been recommended for most PCa patients for decades, especially local late‐stage, metastatic, hormone‐sensitive and castration‐resistant patients.^[^ ^197^ ^]^ However, some patients are less sensitive to ADT. To guide clinical decisions, Bock et al. fabricated a BRM capable of predicting the ADT effect of PCa individuals. As shown in Figure 9Bi,ii, a human osteoblast‐derived mineralized microtissue (hOBMT) was prepared by sequential 3D bioprinting followed by cell culture for 13 weeks. The hOBMT exhibited a typical human osteoblast phenotype (Figure 9Biii), with high vitality (Figure 9Biv), dense ECM/collagen deposition (Figure 9Bv), and both osteoblastic and osteocytic morphologies (Figure 9Bvi). In this model, androgen receptor‐dependent PCa cells exhibited morphological and functional differences under androgen deprivation, while androgen receptor‐independent LNCaP cells displayed clear adaptive responses (Figure 9Bvii). The hOBMT model is superior to the cell line model. In this study, hOBMT enabled the long‐term study of metastatic PCa to quantitatively address hypotheses related to osteoblastic bone metastasis.

BRMs could be further used as patient‐derived models of metastatic UM in vitro. As shown in Figure 9Ci, Shokoohmand et al. seeded human osteogenic precursor cells onto a biocompatible fibrous scaffold and then differentiated them to prepare a tube‐shaped human osteoblastic tissue‐engineered construct (hOTEC). The hOTEC was indirectly cocultured with patient‐derived xenografts (PDXs) of PCa to investigate their molecular interactions (Figure 9Cii). The results showed enhanced mineralization and osteomimicry of hOTEC when cocultured with PDXs derived from lymph node metastasis (LuCaP35) and bone metastasis (BM18) in primary PCa patients. Notably, a research model derived entirely from human primary cells represents a significant advance in the research field of bone tumors.^[^ ^198^ ^]^ Patient‐derived BRMs can serve as a personalized preclinical research platform for investigating the metastasis‐specific molecular mechanisms of UM.

### 4.4 Microfluidic chips

#### 4.4.1 Drug screening

Organ‐on‐a‐chip (OoC) is a modular system in which the physiological functions of tissues and organs are simulated by culturing live cells in a continuously perfused micron‐sized chamber.^[^ ^201^ ^]^ The OoC is defined by three key features: the three‐dimensional and orderly arrangement of different components; the integration of multiple cells (parenchymal cells, stromal cells, vascular cells, and immune cells); and the presence of biomechanical forces (tensile and shear forces).^[^ ^202^ ^]^ Compared to the 2DCC model, the microfluidic OoC is more suitable for drug screening because it allows temporal control of a series of parameters, such as fluid flow, oxygen gradient, and temperature.^[^ ^203^ ^]^ Moreover, the types and dosages of cytokines, growth factors, ECM components related to cell metabolism and cellular signal transduction can be programmed to meet the needs of different applications.^[^ ^204^ ^]^

**FIGURE 10:**
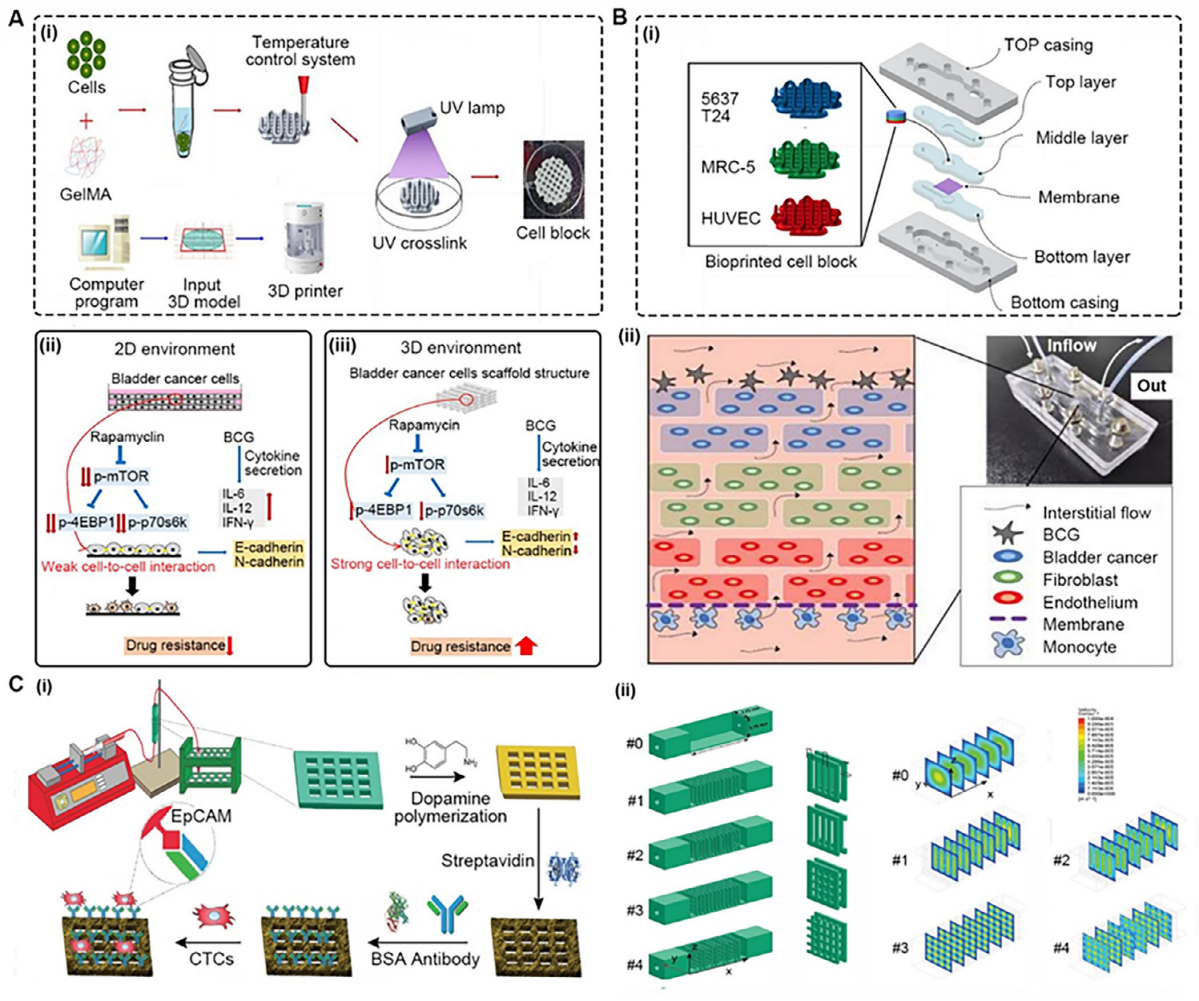
Microfluidic chips for drug screening and isolating circulating tumor cells. (A) A bladder cancer model for characterizing the tumor formation response to chemotherapy: (i) a schematic showing the process of bioprinting and cell culturing; (ii) a comparison of the drug‐resistance effect of bladder cancer cells in 2D and 3D models. Reproduced with permission.^[^ ^219^ ^]^ Copyright 2019, PLOS. (B) A bladder cancer‐on‐a‐chip model for assessing the immunologic reactions response to Bacillus Calmette‐Guérin (BCG): (i) the structure of bladder cancer‐on‐a‐chip model prepared by bioprinting; (ii) a schematic showing the application of the model with the help of the microfluidic system. Reproduced with permission.^[^ ^220^ ^]^ Copyright 2021, MDPI. (C) A microfluidic chip for isolating circulating tumor cells (CTC): (i) a schematic showing the preparation and application of the models; (ii) five kinds of models with different structures were designed, and the velocity magnitude profiles of each model were exhibited. Reproduced with permission.^[^ ^221^ ^]^ Copyright 2020, Elsevier.

BRMs have been used for drug screening for years. As shown in Figure 10Ai, Kim et al. constructed a 3D‐printed, BCa cell‐laden scaffold as a potential drug testing platform for rapamycin and Bacillus Calmette‐Guérin (BCG). In this study, the BRMs (3D environment) exhibited a higher cell proliferation rate and intercellular interactions than 2DCC (2D environment) (Figure A10ii,iii). In conclusion, the patterns of drug resistance in 2D and 3D environments are significantly different. BRMs with unique tumor heterogeneity are superior to 2DCC for drug screening. Currently, BRMs of UM are relatively abundant and can be extensively used for the screening of novel drugs, including plant‐derived drugs, chemotherapy drugs, monoclonal antibodies, and immunosuppressants.

The advantages of OoC and BRMs can be combined. As shown in Figure 10B, Kim et al. developed a BCa chip (BCOC) that integrated bioprinted scaffolds and microfluidic chips. The scaffolds comprised 3 layers, namely, bladder cancer cells, MRC‐5 cells and HUVECs, and formed a structure similar to that of the TME. With the help of microfluidic devices, the scaffolds were cultured with continuous perfusion of culture medium containing BCG. The authors successfully used this model of BCOC to predict the response of individual immune therapy. The cell‐laden scaffolds could be replaced with tumor spheroids and organoids, and a high‐throughput model of BCOC could be developed.^[^ ^205^ ^]^ Notably, a human “body on a chip” multiorgan system has been created by coupling two or more organ chips with fluid to simulate systemic physiology, drug distribution, and configuration.^[^ ^206^ ^]^ Correspondingly, by combining tumor chips with multiple organ systems, a tumor metastasis model of distant tissues and organs can be facilely established to explore more complex tumor patterns.

#### 4.4.2 Isolating circulating tumor cells

Circulating tumor cells (CTCs) are tumor cells that detach from the primary tumor, infiltrate the bloodstream, and circulate in the bloodstream.^[^ ^207^ ^]^ CTCs d with epithelial‐mesenchymal transition (EMT) and stemness characteristics might invade and colonize distal sites, leading to fatal metastasis.^[^ ^208^ ^]^ CTCs are relatively rare, with a frequency of 1 per 10^6^–10^7^ blood cells.^[^ ^209^ ^]^ CTCs are also heterogeneous with considerable genotypic and phenotypic diversity. Currently, the study of CTCs is hampered by a series of obstacles, especially the separation and enrichment of CTCs.^[^ ^210^ ^]^ Researchers have developed both physical and immunological methods to isolate CTCs.

In physical methods, CTCs are isolated according to their cell volume, deformability, density and membrane characteristics. For example, Chunyang Lu et al. reported a microfluidic device integrating focus‐separation speed reduction design and trap arrays for the high‐throughput capture of CTCs.^[^ ^211^ ^]^ In immunological methods, a series of molecular markers, such as EpCAM (prostate cancer, kidney cancer, bladder cancer), PSMA (prostate cancer), and CK‐8, −18, and −19 (prostate cancer, bladder cancer), have been identified. In particular, the Food and Drug Administration (FDA) has approved the clinical application of CellSearch, the only immune affinity‐based platform for separating and counting CTCs.^[^ ^212^ ^]^

Physical methods and immunological methods can be combined into one system to improve the isolation efficiency. As shown in Figure 10Ci, Chen et al. manufactured a microfluidic chip with bioprinting technology and then used it to isolate CTCs from peripheral blood by antibody‐specific interactions. Briefly, the self‐polymerization of dopamine (DA) generated a biocompatible polydopamine (PDA) coating on bioprinted devices in an alkaline solution. The reaction sites on PDA provide accessible covalent anchors for streptavidin, enabling biotinylated anti‐EpCAM antibodies to be immobilized as an interface to capture specific CTCs. The excess active sites are blocked by BSA protein to prevent nonspecific binding. The interactions between tumor cells and the chip could be maximized by increasing the surface area and manipulating fluid flow (Figure 10Cii).

The clinical application of CTCs for the early diagnosis of UM is anticipated. For example, Xiang Ren et al. developed a sequential size‐based microfluidic chip for the separation and detection of CTCs, achieving a capture rate of 95% for PCa cells. Through the optimization of experimental conditions, the viability of the captured PCa cells was maintained, enabling their use for subsequent biological analysis.^[^ ^213^ ^]^ Noninvasive separation and enrichment methods for CTCs have been used in the clinic. For example, CTCs isolated from castration‐resistant PCa patients show positivity for PSA, androgen receptor (AR) splice variant 7 and full‐length AR (AR‐FL), which might be helpful for the diagnosis of PCa.^[^ ^214^ ^]^ Cieślikowski et al. reported that a high CTC count could identify high‐risk PCa with occult metastases.^[^ ^215^ ^]^

CTCs have been less explored in UMs than PCa, partly owing to their low positive rate. For example, Soave et al. reported CTCs in only 21.3% of 141 BCa patients before surgery.^[^ ^216^ ^]^ To improve the detection ability, captured CTCs could be analyzed in depth by a combination of RNA‐seq and single‐cell RT‒PCR.^[^ ^217^ ^]^ Longitudinal sampling and monitoring of tumor progression before and after specific treatments could be performed in the same way.^[^ ^218^ ^]^ Notably, the isolation of CTCs and the early diagnosis of UM are conducted in a laboratory environment. The clinical translational guidelines for diagnostic reagents and devices are relatively loose. In the near future, the CTC‐based early diagnosis of UM will be extensively used in clinical practice.

## 5 CONCLUSION AND PERSPECTIVES

UM is a vigorous field that has recently undergone a series of landmark advances, including mechanistic investigation, early diagnosis, precision medication, immunotherapy, and nanomedicine.^[^ ^222^ ^]^ BRMs are considered one of the frontiers of interdisciplinary science and have the potential to disrupt the paradigms of traditional research, including UM.^[^ ^223^ ^]^ An increasing number of BRMs of UM are being reported in the literature. Six kinds of bioprinting techniques, EBB, DBB, PBP, LDWB, M3D and ADP, can be used to fabricate such BRMs. Notably, several crucial factors in BRMs, including the chemical composition and spatial structure and the harvest and culture of seeding cells, can be optimized according to different application scenarios. BRMs of UM have been successfully applied for culturing tumor spheroids and organoids, modeling cancer metastasis, mimicking the tumor microenvironment, constructing organ chips for drug screening and isolating circulating tumor cells. The emergence of BRMs with programmed spatial structures and properties as a powerful research tool has greatly spurred fundamental research on UM.

Despite milestone achievements, there are some obstacles to the development and application of bioprinting. For example, creating fine features at the cellular level within a desirable bioprinting volume are necessary to support the fabrication of complex and functionalized BRMs.^[^ ^224^ ^]^ However, limited spatial resolution remains a key bottleneck of most bioprinting techniques. To solve this problem, high‐definition (HD) bioprinting was recently proposed, defined as the fabrication of 3D constructs with feature sizes below 50 μm using cell‐laden bioinks.^[^ ^225^ ^]^ HD bioprinting is rapidly improving the resolution, mainly by decreasing the thickness of the printed layer, pixel size, and single line size and by developing novel tomographic reconstruction algorithms (TRAs) with improved contrast and light distribution. In recent years, a series of advanced bioprinting techniques, including multimaterial bioprinting (MMB), 4D bioprinting and artificial intelligence (AI)‐assisted bioprinting, have attracted increasing interest. MMB is defined as the printing of two or more polymer‐based inks in a programmed manner to form one system with region‐specific features and performances.^[^ ^226^ ^]^ Each polymer‐based ink can be single or multicomponent, single phase or composite. MBB is desirable for fabricating BRMs with complex constructs, especially tumor microenvironments. 4D bioprinting refers to the fabrication of bioprinted constructs with structures, properties, and bioactivities that can evolve over time with or without a predetermined stimulus.^[^ ^227^ ^]^ 4D bioprinting is suitable for fabricating smart, stimulus‐responsive BRMs for drug discovery and nanomedicine. AI‐assisted bioprinting is a brand‐new concept. AI can self‐learn massive structural relationships without rule‐based programming or user commands.^[^ ^228^ ^]^ For example, Chen et al. developed an AI‐assisted system for the high‐throughput screening of the experimental conditions for bioprinting.^[^ ^229^ ^]^ The benefits of AI include but are not limited to improved ink design, optimized printing conditions, identification of the relationships between structure‐bioactivity and ink‐hosts, and pattern recognition of emerging biological behavior. AI is expected to further improve the spatial structure and function of BRMs, thus improving the effectiveness of their application by better simulating the physiological TME. More information about bioprinting techniques was summarized in our previous review.^[^ ^105b^ ^]^

Improving the viability of seeding cells during the process of bioprinting is another challenge of bioprinting.^[^ ^230^ ^]^ Cell viability is affected by stress, including that caused by environmental factors, such as pH and temperature, and the effect becomes more severe as the intensity and duration of stress increase. Several potential strategies to protect seeding cells from bioprinting injuries are summarized in a relevant review.^[^ ^231^ ^]^ Researchers are exploring additional potential seeding cells to improve the abundance and function of BRMs in order to meet the emerging and challenging needs of fundamental research on UM. The most commonly used seeding cells are immortalized tumor cells, which are easy to cultivate in vitro but lack tumor heterogeneity.^[^ ^232^ ^]^ Primary tumor cells can maintain their genetic characteristics in vivo and better model the growth status of tumors; thus, they are more suitable for experimental investigation.^[^ ^233^ ^]^ Primary tumor cells are isolated from patient tumors. The establishment of a clinical biobank and the harvest and culture of primary cells, especially conditionally reprogrammed cells (CRCs) of bladder cancer, have been comprehensively reviewed in our previous works.^[^ ^105^ ^]^ Multimaterial bioprinting has been used to print multiple cells, such as tumor cells, stromal cells, and immune cells, into one system to obtain research models that simulate the characteristics of the TME. The model will provide a powerful tool for the large field of tumor immunity research. Currently, the harvest and culture of seeding cells still faces some challenges, including a lack of tissue donors, low yield, high cost, poor consistency, and high time consumption.^[^ ^234^ ^]^ The induced differentiation of stem cells is one of the potential approaches to solve these problems and will require further assistance from developmental biology.

The applications of BRMs will be greatly expanded in the upcoming interdisciplinary era. For example, antitumor biomaterials, developed by materials science, are regulated by the Food and Drug Administration (FDA, USA) and the National Medical Products Administration (NMPA, China). According to the general guidelines of the FDA and the NMPA, a series of preclinical research models, such as tumor‐bearing models, primary tumor models, metastatic tumor models, recurrent tumor models, drug‐resistant tumor models, and humanized tumor models, with or without immunodeficiency, are urgently needed for evaluating the effectiveness and safety of these antitumor biomaterials before clinical translation. BRMs of UM can effectively meet these needs. It is remarkable that humanized tumor spheroids prepared by bioprinting not only can be used in vitro but also can be further transplanted into animals to obtain a humanized graft model, which is significantly different from the conventional patient‐derived xenograft (PDX) model.^[^ ^235^ ^]^ Herein, it is strongly recommended to enhance the connection between BRMs and the clinical data of UM. In particular, tissue samples from patients and their families with rare genetic and pathological characteristics are valuable for precision medicine, and they should be collected and processed into BRMs for systematic study.

In conclusion, the exploration of BRMs of UM is increasing. The preparation and application potential of these BRMs remain to be revealed. Several cutting‐edge techniques, including 4D bioprinting and artificial intelligence (AI)‐assisted bioprinting, will greatly improve BRMs and fundamental research on UM. Thus, we call for researchers to devote more attention to this field.

## CONFLICT OF INTEREST STATEMENT

The authors declare no conflicts of interest.

## Data Availability

The data that support the findings of this study are available from the corresponding author upon reasonable request.
